# Epigenetic silencing of *DLEC1* correlates with tumor immune microenvironment and predicts immunotherapy prognosis in multiple cancers

**DOI:** 10.1007/s00439-026-02828-3

**Published:** 2026-04-20

**Authors:** Ruijie Ming, Qi Xiong, Shuh-Ying Tan, Lili Li, Shujie Bao, Jianhua Wang, Lijuan Zhao, Xiaoqian He, Zeze Zheng, Yan Wang, Xiaoyu Liu, Jun Tang, Zhongjun Wu, Tingxiu Xiang, Qian Tao

**Affiliations:** 1 Department of Oncology, The First Affiliated Hospital of Chongqing Medical University, Chongqing, 400030 China; 2 Chongqing Key Laboratory for the Mechanism and Intervention of Cancer Metastasis, Chongqing University Cancer Hospital, Chongqing University, Chongqing, 400030 China; 3 Johns Hopkins Singapore, Singapore, Singapore; 4 Cancer Epigenetics Laboratory, Department of Clinical Oncology, State Key Laboratory of Translational Oncology and Sir YK Pao Center for Cancer, The Chinese University of Hong Kong, Hong Kong, Hong Kong SAR China

## Abstract

Cancer remains a major health issue globally, with increasing incidence and mortality rates. While immunotherapy has revolutionized cancer treatment, not all patients benefit, urging the identification of predictive biomarkers. This study utilized public datasets and tumor samples to examine the expression and promoter hypermethylation of *DLEC1* in normal and tumor tissues, to evaluate its potential as prognostic and immunotherapy marker. *In vitro* and *in vivo* experiments on breast cancer (BrCa) cell lines were conducted to explore the functions and mechanisms of *DLEC1*. The prognostic significance of *DLEC1* downregulation and its impact on immune cell infiltration and immunotherapy efficacy were analyzed using the GEO and TCGA databases. *DLEC1* was frequently downregulated and methylated across multiple cancers. In BrCa, expression of *DLEC1* inhibited tumor cell proliferation, induced apoptosis, and activated the interferon lambda1 signaling pathway, indicating its important role as a *bona fide* tumor suppressor and immunoregulator. High *DLEC1* expression correlated with better prognosis in several cancer types and with increased immune cell infiltration. Cancer patients with high *DLEC1* expression showed improved overall and progression-free survival under immune checkpoint inhibitor therapies, such as anti-CTLA4, anti-PD1 and anti-PDL1. *DLEC1* promoter hypermethylation is a promising biomarker for cancer prognosis and potential immunotherapy response prediction. Its expression and promoter hypermethylation status could thus help to guide clinical treatment strategies for cancer patients, particularly of breast cancer.

**Supplementary Information:**

The online version contains supplementary material available at 10.1007/s00439-026-02828-3.

## Introduction

Cancer remains a significant public health challenge globally, with incidence and mortality rates rising annually (International Agency for Research on Cancer). In 2022, there were ~ 20 million new cases and 9.7 million cancer-related deaths (Bray et al. 2024). Traditional cancer treatments include surgery, radiotherapy, chemotherapy, and targeted therapy (Yahya and Alqadhi 2021). In recent years, immunotherapy, particularly immune checkpoint inhibitors, has emerged as a frontline treatment for certain cancers (Larroquette et al. 2021, Akhbariyoon et al. 2021). However, not all cancer patients benefit from immunotherapy (Donisi et al. 2024), urging the need to develop biomarkers to identify and classify patients likely to respond to immunotherapy. The development of this type of predictive biomarkers is crucial for optimizing clinical treatment strategies and improving outcomes for cancer patients.

Deleted in Lung and Esophageal Cancer 1 (*DLEC1*) is located on the short arm of chromosome 3 (3p22-p21.3) and encodes a 1755-amino acid polypeptide (Daigo et al. 1999, Rauch et al. 2006). Allelic loss in this region is a common and significant event in the pathogenesis of multiple cancers (Senchenko et al. 2003, Naylor et al. 1987, Kovacs et al. 1988, Ogasawara et al. 1995, Huang et al. 1991, Sato et al. 1991). *DLEC1* expression is often downregulated or silenced due to promoter CpG hypermethylation in various cancers, including lung (Rauch et al. 2006), ovarian (Kwong et al. 2006), nasopharyngeal (Kwong et al. 2007), gastric (Kang et al. 2008), renal cell carcinoma (Zhang et al. 2010), breast (Park et al. 2011), lymphoma (Wang et al. 2012) and prostate cancers (Zhang et al. 2015). Restoration of *DLEC1* expression inhibits the proliferation of hepatocellular carcinoma cells and induce G1 phase cell cycle arrest (Qiu et al. 2008), suggesting that *DLEC1* may play diverse regulatory roles in cancer development, including tumor cell proliferation and migration. However, comprehensive studies exploring the prognostic and immunotherapeutic prognostic value of *DLEC1* across various cancer types are still lacking.

In this study, we leveraged multiple public datasets and collected tumor samples to elucidate the expression and promoter methylation levels of *DLEC1* in normal and tumor tissues. We also investigated the potential functions of *DLEC1* and its impact on immune cell infiltration. Using survival data from the GEO and TCGA, we examined the prognostic significance of *DLEC1* and its potential influence on immune checkpoint therapy responses. Finally, through in vitro and in vivo experiments on breast cancer cell lines, we explored the role of *DLEC1* and discovered that its mechanisms involve immune-related pathways. Our systematic analysis suggests that *DLEC1* is a promising biomarker for prognosis and potentially associated with immunotherapy responses.

## Materials and methods

### Analysis of expression and promoter methylation levels of *DLEC1*

To minimize batch effects and ensure comparability between datasets, *DLEC1* mRNA expression data for both normal (the Genotype-Tissue Expression Project database, GTEx) and tumor (The Cancer Genome Atlas, TCGA) tissues were obtained from the UCSC Xena Toil RNA-seq Recompute dataset. This dataset applies a unified data processing pipeline (STAR alignment and RSEM quantification) to both cohorts. The expression values were quantified as Transcripts Per Million (TPM) and normalized using log2(TPM + 1) transformation. Information on *DLEC1* mRNA expression and promoter methylation in normal and tumor tissues, alongside clinical data of cancer patients, was sourced from TCGA database using the R package *TCGAbiolinks* (Colaprico et al. 2016). To cross-validate the methylation patterns observed in our primary TCGA analysis, we utilized the MethHC database (http://awi.cuhk.edu.cn/~MethHC/methhc_2020/php/index.php) (Huang et al. 2021), which serves as an independently processed and curated repository of TCGA methylation data. The R package *limma* was utilized to analyze differences in *DLEC1* mRNA expression and promoter methylation between normal and tumor tissues (Ritchie et al. 2015), and results were visually represented using the R package *ggplot2* (Wickham 2016).

### Human sample collection and RT-PCR

Breast tissue samples were obtained from the Department of Oncology and Breast Surgery at the First Affiliated Hospital of Chongqing Medical University, China. Each sample underwent histological evaluation and diagnosis by expert pathologists. Informed consent was secured from all participants. Prior to research use, samples were preserved in the tissue bank at Chongqing Medical University. The study received approval from the Institutional Review Board of Chongqing Medical University (Approval notice: # 2016-75).

Reverse transcription utilized Promega GoScript reverse transcriptase (Promega, USA). RT-PCR was conducted using Go-Taq (Promega, USA) and the GeneAmp RNA PCR system (Applied Biosystems, USA), with GAPDH serving as the internal reference. Quantitative reverse transcription PCR (qRT-PCR) was performed using SYBR Green (Thermo Fisher, USA) on the LightCycler real-time PCR system (Roche, CH), also referencing GAPDH. All primer sequences were detailed in Table S1.

### Methylation-specific PCR (MSP) and bisulfite genomic sequencing (BGS)

Genomic DNA was extracted from tissues and cell lines using the QIAamp DNA Mini Kit (Qiagen, GER). DNA bisulfite treatment followed previously published protocols (Tao et al. 2002, Tao et al. 1999). The promoter methylation status of *DLEC1* was evaluated within the CpG island spanning the promoter and exon 1 region. MSP and BGS were performed to detect the promoter methylation levels. The specific primer sequences are listed in Table S1 (Zhang et al. 2015). All primers were validated to ensure they did not amplify untreated DNA. PCR products were evaluated on 2% agarose gels.

BGS utilized primers (Table S1) to amplify bisulfite-treated DNA. The PCR products were cloned into a vector (Invitrogen, USA), with 8–12 colonies randomly selected for sequencing.

### Patient samples

A cohort of 140 primary breast cancer patients was recruited at the First Affiliated Hospital of Chongqing Medical University. From these patients, a total of 150 primary breast tumor tissue samples were obtained during surgical resection and analyzed for *DLEC1* promoter methylation status (results in Supplementary Table S2). Crucially, none of the enrolled patients received neoadjuvant chemotherapy or radiotherapy prior to surgery. The clinicopathologic characteristics of the 140 patients are summarized in Supplementary Table S3. Additionally, 4 normal breast tissues were included as negative controls for the methylation assay. All patients provided informed consent, and this study was approved by the Institutional Ethics Committee of the First Affiliated Hospital of Chongqing Medical University.

### Pan-cancer prognostic analysis of *DLEC1*

*DLEC1* expression and survival data from the TCGA database were accessed through R package *TCGAbiolinks* and UCSC Xena (http://xena.ucsc.edu/). Overall survival (OS), disease-free survival (DFS), disease-specific survival (DSS), and progression-free survival (PFS) analyses assessed *DLEC1*’s prognostic and prognostic values across cancers. Kaplan-Meier analysis was carried out using the R packages *survminer* and *survival* (Biecek 2021, Terry and Therneau 2000). Patients in each cancer type were categorized into high and low *DLEC1* groups based on OS cutoff values. The KM-plot online tool subsequently examined post-progression survival (PPS) and first progression (FP) in BRCA, LUAD, and STAD patients (Győrffy 2024).

### Immune score and immune cell infiltration analysis

The correlation between *DLEC1* and immune scores, including stromal and ESTIMATE scores, was analyzed across TCGA pan-cancer datasets using the R package *TCGAplot* (Liao and Wang 2023). The correlation between *DLEC1* expression and the abundance of immune infiltrates was analyzed using the TIMER2.0 database (Li et al. 2020). To account for the potential confounding effect of tumor purity, we utilized the ‘Purity Adjustment’ module to calculate partial Spearman’s correlations (R). For multiple comparisons, we utilized the FDR-adjusted p-values provided by the TIMER2.0 web server. Statistical significance was defined as an FDR < 0.05.

### Anticancer immune response analysis

The Tracking Tumor Immunophenotype (TIP) database (http://biocc.hrbmu.edu.cn/TIP) was utilized to analyze the impact of *DLEC1* expression on anticancer immune responses across 33 cancer types (Xu et al. 2018). Kendall’s correlation test was performed to evaluate the association between *DLEC1* expression and immune activity scores across the seven steps of the cancer-immunity cycle. To control for multiple comparisons, p-values were adjusted using the Benjamini-Hochberg (BH) False Discovery Rate (FDR) method. Statistical significance was categorized as follows: * FDR < 0.05, ** FDR < 0.01, and *** FDR < 0.001. The results were graphically represented using the R package *ggplot2* (Wickham 2016).

### Prediction of immunotherapy response

The correlation between *DLEC1* and immunomodulatory genes (analyzing the pre-defined immune-related gene sets provided by the package, including immunostimulators and immunoinhibitors) was obtained through R package *TCGAplot* (Liao and Wang 2023). Tumor mutation burden (TMB) and microsatellite instability (MSI) data for 33 cancer types were downloaded from UCSC Xena, and the correlation between *DLEC1* expression and TMB, MSI was analyzed through the cor.test function (spearman method) and graphically displayed by the R package *fsmb* (Nakazawa 2024). The KM-plot online tool was used to evaluate the effect of *DLEC1* expression on the prognosis of tumor patients treated with immune checkpoint inhibitors (specifically anti-PD-1, anti-PD-L1, and anti-CTLA-4 cohorts), including OS and PFS (Sample information provided in Table S4; Kovács et al. 2023).

### Cell Lines, plasmids, and construction of DLEC1 overexpression cell lines

Breast cancer cell lines (BT549, MDA-MB-231, MCF-7, T-47D, YCC-B1, YCC-B3, ZR-75-1, MDA-MB-468, SK-BR-3) were purchased from ATCC or provided by collaborators. Cells were cultured in RPMI 1640 medium (Gibco-BRL, GER) supplemented with 10% fetal bovine serum (FBS, Gibco-BRL, GER), 100 U/mL penicillin and 100 mg/mL streptomycin (Gibco-BRL, GER) and incubated at 37 °C in 5% CO_2_. HEK293T cells were cultured in DMEM medium (high glucose, Gibco-BRL, GER) with 10% FBS. The pcDNA3.1-DLEC1 plasmid was constructed by cloning the full-length *DLEC1* coding sequence into pcDNA3.1 vector with sequence verified.

Lipofectamine 2000 (Invitrogen, USA) were used for transfection following the manufacturers’ instruction. MDA-MB-231 and BT549 were transfected with DLEC1 plasmids and filtrated with G418 to establish stable overexpressing-DLEC1 cell lines. The pcDNA3.1- empty plasmid was transfected into generated control cell lines. RT-PCR was performed to confirm overexpression of DLEC1.

### Drug demethylation

Cells were treated with 10 mM azacitidine (Aza, Sigma-Aldrich, USA) for 72 h, followed by an additional treatment with 100 nM trichostatin A (TSA, Cayman Chemical, USA) for 24 h to demethylation. DNA was then extracted and subjected to MSP as above described.

### Western blot

Protein was extracted from transfected cells 48 h after transfection with DLEC1 plasmid or empty vector control. Then the cell lysate was separated by sodium dodecyl sulfate polyacrylamide gel electrophoresis (SDS-PAGE), and then transferred to polyvinylidene fluoride (PVDF) membrane. After blocked with 5% milk in TBST for 1 h, the membrane was incubated with the primary antibody of DLEC1 (#20027-1-AP, Proteintech, USA). Then, the membrane was incubated in the second antibody solution, and the western blot was displayed using the enhanced chemiluminescence detection system. GAPDH (ab263962, Abcam, USA) was used as control.

### Cell proliferation assays

A total of 200 cells were seeded into new 6-well plates and cultured in fresh medium for two weeks to inspect the ability of clone formation, as detailed previously (Wang et al. 2019). Cell proliferation was measured at 0, 24, 48, and 72 h using the Cell Counting Kit-8 (CCK8, Beyotime, China), as previously described (Wang et al. 2019). Each experiment was repeated three times.

### Flow cytometry

Cell cycle and apoptosis were analyzed by flow cytometry (FCM) (Li et al. 2014). Cells were fixed with precool absolute ethanol at −20 °C, stained with propidium iodide (PI) for cell cycle detection, and with annexin V-fluorescein isothiocyanate and PI for apoptosis detection. The CellQuest kit (BD Biosciences, USA) was used to evaluate FCM results. All experiments were repeated three times.

### Transwell assays for migration and invasion

Cell migration was assessed using Transwell chambers with 6.5 mm diameter and 8 μm pore size (Corning Incorporated, USA). For invasion assays, Transwell membranes were coated with Matrigel (BD Biosciences, USA). Cells on the lower surface of the chamber were fixed and stained after 24 h, and images were captured using a phase contrast microscope (Leica, GER), followed by cell counting. All experiments were repeated three times.

### Subcutaneous xenograft model

All procedures and experimental protocols involving mice were approved by the Animal Center of Chongqing Medical University. Six 4–6-week-old BALB/c nude mice were used for the experiments. MDA-MB-231 cells with or without DLEC1 overexpression were digested to generate cell suspensions. Each mouse was subcutaneously injected with 200 µl PBS containing 2.5 × 10^6 cells. Primary tumor size was measured every two days starting seven days post-injection. The maintenance of the housing facility adhered to national standards (Laboratory Animal-Requirements of Environment and Housing Facilities; GB 14925 − 2010). Experimental animal management and operations complied with the “Chongqing City Experimental Animal Management Measures” (Chongqing Government Order No. 195). Tumor volume (mm^3) was calculated using the formula: Volume = Length × Width^2 × 0.52.

### Immunohistochemistry

Immunohistochemistry (IHC) was used to detect protein expression in tissues. Collected tissues were sectioned into 4 μm slices, baked overnight at 65 °C, washed with PBS, and stained using an immunohistochemical staining kit (BIGB-BIO, China) according to the manufacturer’s instructions. Slides were incubated overnight (16–20 h) at 4 °C with primary antibodies against Ki67 (#16667, Abcam, USA) and DLEC1 (#20027-1-AP, Proteintech, USA). The following day, slides were stained with DAB substrate (K176810 E, China) for 30 s and counterstained with hematoxylin for 3 s. Images were examined under a microscope.

### Cytokine chip

The supernatant from breast cancer cells with stable overexpression of DLEC1 and control cells, after 24 h of culture, was collected. A cytokine chip (CodePlex, isoplexis, GER) was used to detect 22 cytokines associated with human adaptive immunity. These cytokines include GM-CSF, Granzyme B, IFN-γ, IL-2, IL-4, IL-5, IL-6, IL-7, IL-8, IL-9, IL-10, IL-13, IL-15, IL-17A, IP-10, MCP-1, MIP-1α, MIP-1β, Perforin, sCD137, TNF-α, and TNF-β. The experiment was conducted following the operational protocol provided.

### Data analysis

Statistical analyses were conducted using SPSS (23.0, USA), R (4.3.3) and GraphPad Prism (8.0.1, USA) software. Key R packages used in this study included *TCGAbiolinks* (version 2.28.3), *limma* (version 3.58.1), *survival* (version 3.5–8.5), *survminer* (version 0.4.9), *TCGAplot* (version 7.0.1), and *fsmb* (version 0.7.6). Web-based resources were accessed as follows: TIMER2.0 (http://timer.cistrome.org/) was accessed on May 2024, and the TIP database (http://biocc.hrbmu.edu.cn/TIP) was accessed on May 2024. For comparisons of continuous variables, two-tailed Student’s t-test was used for normally distributed data, and the Mann-Whitney U test was used for non-normally distributed data. Chi-square test and Fisher’s exact test were used for comparisons of categorical data. To account for multiple hypothesis testing in high-throughput data analysis (including differential expression analysis and clinical stage comparisons), p-values were adjusted using the Benjamini-Hochberg (BH) method to control the False Discovery Rate (FDR); an FDR < 0.05 was considered statistically significant. For exploratory analyses involving pan-cancer correlations (e.g., TMB, MSI, and immune infiltration) visualized using the *TCGAplot* package, raw p-values (Spearman’s correlation or Wilcoxon test) were reported to visualize potential trends without multiple hypothesis correction, unless otherwise specified. The Kaplan-Meier method was used to evaluate the impact of DLEC1 levels on prognosis. Kendall correlation analysis was employed to analyze the correlation between DLEC1 and anticancer immune response. Spearman correlation analysis was used to examine the relationship between the expression of DLEC1 and immune-related regulatory genes. Statistical significance was defined as FDR or *p* < 0.05, where applicable.

## Results

### Expression and methylation patterns of *DLEC1* in pan cancers

**Fig. 1:**
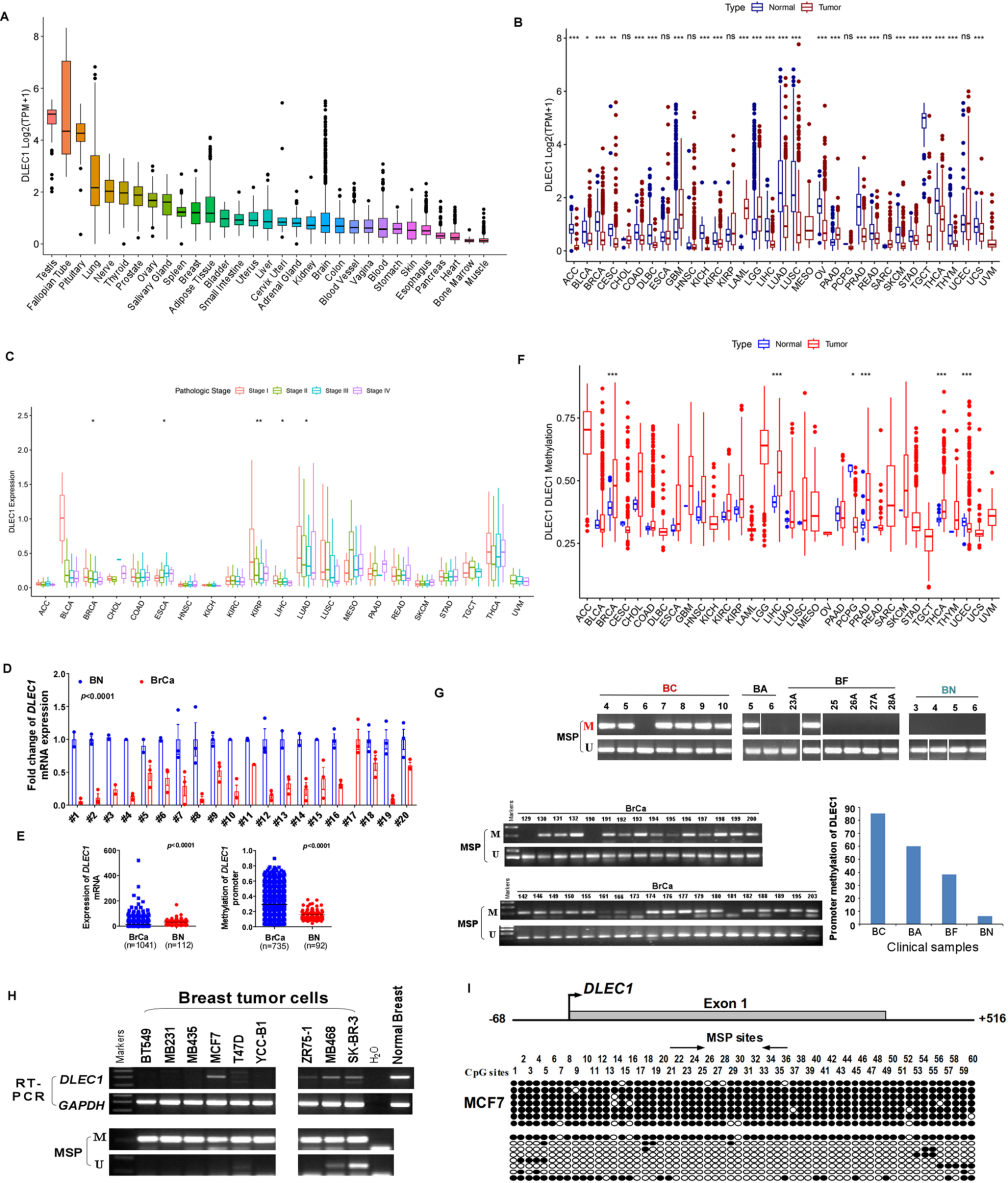
DLEC1

We used the GTEx database to investigate the baseline expression levels of *DLEC1* in normal tissues. As shown in Fig. 1A, *DLEC1* is relatively highly expressed in tissues such as the testis, fallopian tube and pituitary, while it has lower expression in the heart, bone marrow and muscle. To compare the differences in *DLEC1* mRNA levels between cancerous and normal tissues, we downloaded expression data from the TCGA and GTEx databases (incorporating GTEx to increase the sample size of normal controls).

The analysis revealed that *DLEC1* is significantly downregulated in most cancer tissues compared to normal tissues, except for CHOL, ESCA, GBM, KIRP, LAML, and LGG (Fig. 1B). Additionally, in certain tumors such as BRCA, LIHC, LUAD, and LUSC, DLEC1 expression further decreases with increasing stages (Fig. 1C). In the MethHC database, the expression level of *DLEC1* in breast cancer was significantly lower than that in normal breast tissue (Figure S1A). Quantitative RT-PCR results from our collected breast cancer samples also showed that *DLEC1* expression levels were significantly higher in adjacent normal tissues compared to breast cancer tissues (Fig. 1D). In TCGA-BRCA cohort, *DLEC1* was also significantly overexpressed and hypomethylated in tumor tissues (Fig. 1E).

To explore the promoter methylation status of *DLEC1* in tumor and normal tissues, we downloaded 450K methylation data from the TCGA database. As shown in Fig. 1F, DLEC1 is highly methylated in several cancers, including BRCA, CHOL, HNSC, KIRC, KIRP, LIHC, PRAD and THCA. Similarly, in the MethHC database, *DLEC1* is significantly hypermethylated in breast cancer tissues compared to normal breast tissues (Figure S1B). DLEC1 exhibits promoter hypermethylation and low expression in most breast cancer cell lines (Fig. 1H). In our collected breast cancer samples, DLEC1 also showed promoter hypermethylation (Fig. 1G).

Additionally, we observed that the methylation ratio of the CpG island in both promoter and exon1 regions of *DLEC1* decreases with increasing distance from breast cancer tissue (Fig. 1G and Figure S1C-H). The detailed methylation ratios and the corresponding clinicopathological characteristics of patients with breast cancer were shown in Table S2 and Table S3. Upon treatment with Aza for demethylation, the expression levels of *DLEC1* in breast cancer cell lines showed a significant recovery (Fig. 1I, Figure S1I-J), which indicated that demethylation can restore *DLEC1* expression, further emphasizing the role of promoter hypermethylation in regulating *DLEC1* expression in breast cancer. This indicated that *DLEC1* maybe downregulated expression cause promoter hypermethylation. And there was a significant correlation between *DLEC1* expression and promoter hypermethylation status in most tumors (Figure S2).

### DLEC1 restoration inhibits malignant phenotype of breast cancer cells

**Fig. 2:**
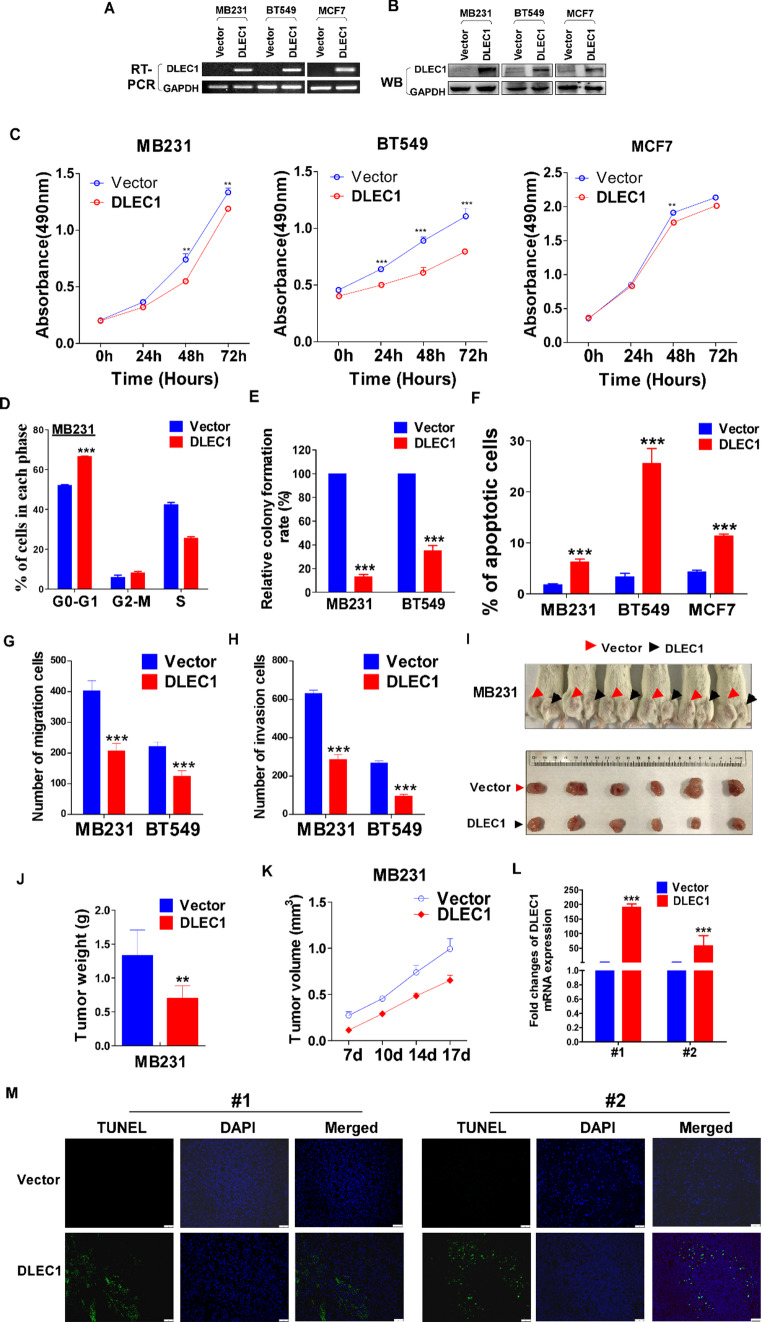
Overexpression of DLEC1 inhibited the malignant phenotype of breast cancer cell lines. **A** Verification of DLEC1 overexpression by RT-PCR. **B** Verification of DLEC1 overexpression by western blot. **C** Proliferation assays showing inhibition of MDA-MB-231, BT549 and MCF-7 cells with DLEC1 overexpression. **D** Cell cycle percentage detected by flow cytometry. **E** Colony formation assays showing reduced colony-forming ability in DLEC1 overexpressing cells. **F** Flow cytometry analysis showing increased apoptosis in DLEC1 overexpressing cells. **G**, **H** Transwell assays showing inhibited migration and invasion in DLEC1 overexpressing cells. **I**–**L** In vivo proliferation inhibition in DLEC1 overexpressing MDA-MB-231 cells. **M** TUNEL staining showing apoptosis

To further investigate DLEC1’s role in tumor cells, we established stable DLEC1 overexpressing MDA-MB-231, BT549 and MCF-7 breast cancer cell lines, verified through RT-PCR and western blot (Fig. 2A-B). CCK8 and cell proliferation assays demonstrated that DLEC1 overexpression significantly inhibited the proliferation of MDA-MB-231, BT549 and MCF-7 cells (*p* < 0.05, Fig. 2C). Colony formation assays showed that DLEC1 overexpression significantly reduced the colony-forming ability of MDA-MB-231 and BT549 cells (*p* < 0.001, Fig. 2E, Figure S3A). Flow cytometry results indicated that DLEC1 overexpression caused G_0_/G_1_ phase arrest in MDA-MB-231 cells and significantly increased the apoptosis rate in MDA-MB-231 and BT549 cells (*p* < 0.001, Fig. 2F, Figure S3B-C). Transwell assays showed that DLEC1 overexpression significantly inhibited the migration and invasion abilities of MDA-MB-231 and BT549 cells (*p* < 0.001, Fig. 2G-H, Figure S3D-E).

Xenograft experiments indicated that DLEC1 overexpression significantly inhibited in vivo proliferation of MDA-MB-231 cells (*p* < 0.01, Fig. 2I-K). IHC analysis of tumors harvested from mice showed that Ki67 expression was markedly reduced in DLEC1 overexpressing MDA-MB-231 cells, indicating decreased proliferation (Fig. 2L, Figure S3F-G). TUNEL staining revealed increased apoptosis in DLEC1 overexpressing MDA-MB-231 cells (Fig. 2M).

#### Overexpression of DLEC1 activated the IFNL1-related pathway in breast cancer cell lines

**Fig. 3:**
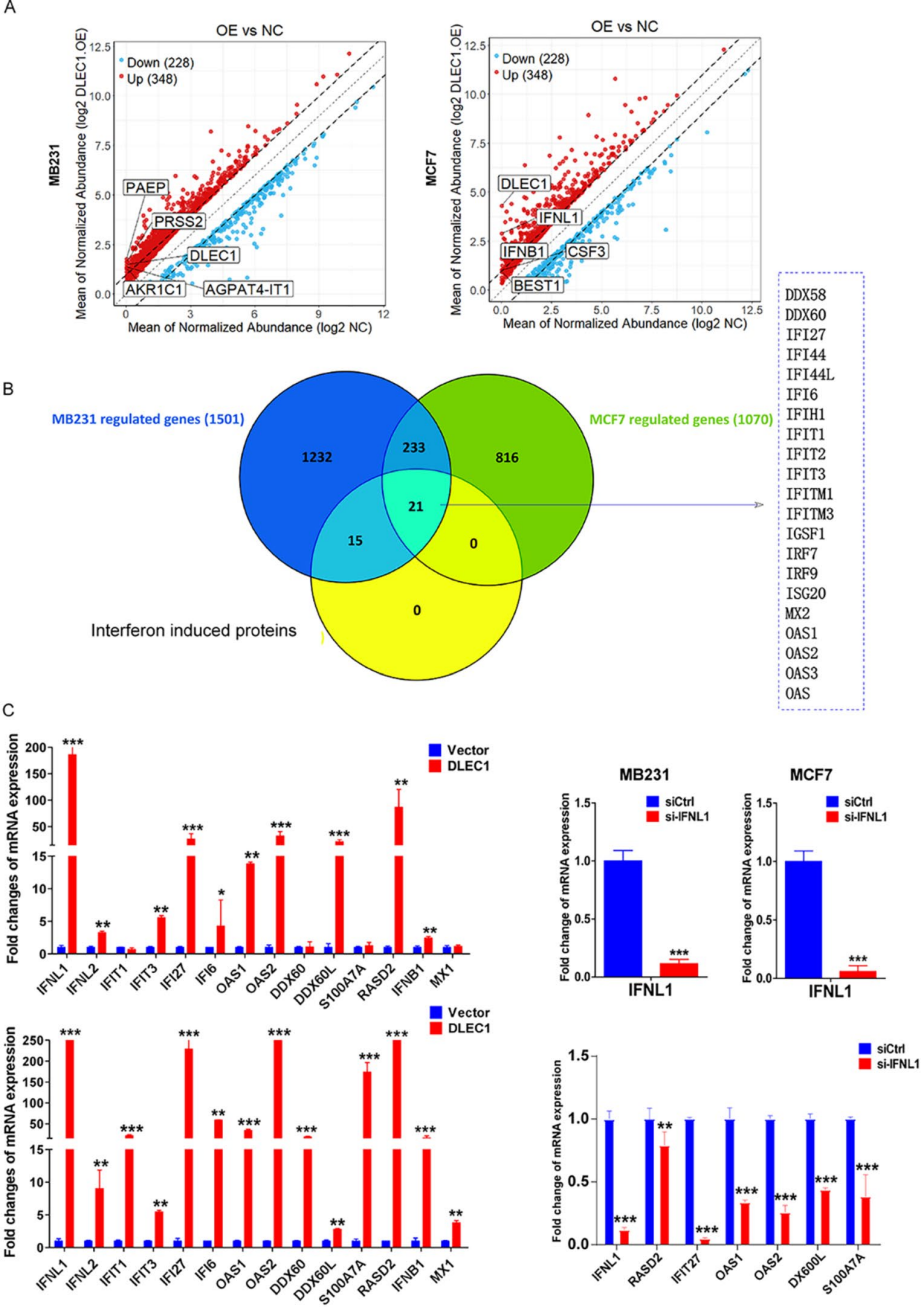
**A**, **B** RNA sequencing showing elevated interferon induced proteins regulated by DELC1. **C** qRT-PCR results confirming upregulated expression of interferon λ1, λ2, and related genes. The right panel shows the rescue experiment where IFNL1 was knocked down using siRNA in DLEC1 overexpressing cells, leading to the downregulation of downstream interferon-stimulated genes

RNA sequencing of DLEC1 overexpressing MCF-7 cells and control cells revealed significantly elevated mRNA levels of interferon λ1 and β1 (Fig. 3A). And it was shown that DLEC1 could regulate interferon-related pathway production (Fig. 3A-B). Subsequent qRT-PCR results confirmed that DLEC1 overexpression significantly upregulated the expression of interferon λ1, λ2, and related genes in MDA-MB-231 and BT549 cells (*p* < 0.05, Fig. 3C). To confirm causality, we performed a rescue experiment by transfecting IFNL1-siRNA into these DLEC1 overexpressing cells. As expected, silencing IFNL1 significantly reversed the upregulation of downstream interferon-related genes (*p* < 0.05, Fig. 3C), indicating that DLEC1 modulates these immune signatures via the IFNL1 pathway.

#### Prognostic value of *DLEC1* in human tumors

**Fig. 4:**
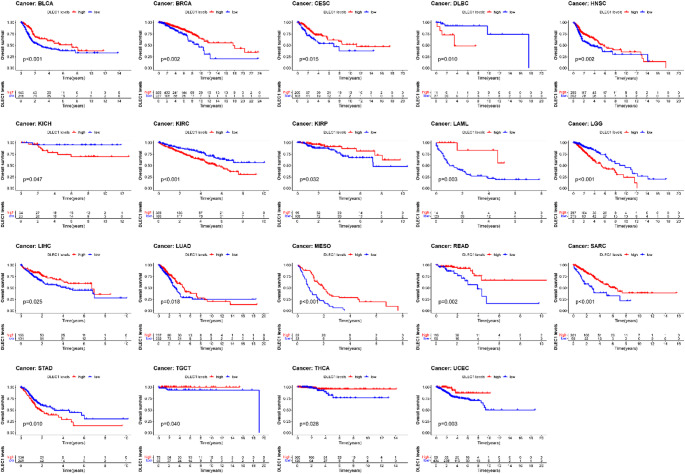
Kaplan-Meier survival curves of cancer patients with different *DLEC1* expression

We assessed the prognostic value of *DLEC1* expression in tumors by analyzing data from TCGA database, which included *DLEC1* expression levels and clinical information of cancer patients. It was observed that patients with high *DLEC1* expression had significantly better prognosis compared to those with low *DLEC*1 expression in BLCA, BRCA, CESC, HNSC, KIRP, LAML, LIHC, LUAD, MESO, READ, SARC, TGCT, THCA AND UCEC (Fig. 4). Conversely, in another 5 tumors, high *DLEC1* expression was associated with poorer prognosis (Fig. 4). These findings were further supported by analysis of DFS, DSS and PFS (Figure S4-6).

To further validate the impact of *DLEC1* expression on tumor prognosis, we utilized multiple GEO datasets. Kaplan-Meier survival analysis consistently showed that in BRCA and LUAD, patients with high *DLEC1* levels had better post-progression survival (PPS) and first progression (FP). Similarly, in STAD patients, high *DLEC1* levels were consistently risk factors for FP and PPS (Figure S7).

Overall, these results indicated that *DLEC1* expression level was an effective prognostic factor for multiple types of cancer.

#### Impact of *DLEC1* expression on tumor immune cell infiltration

**Fig. 5:**
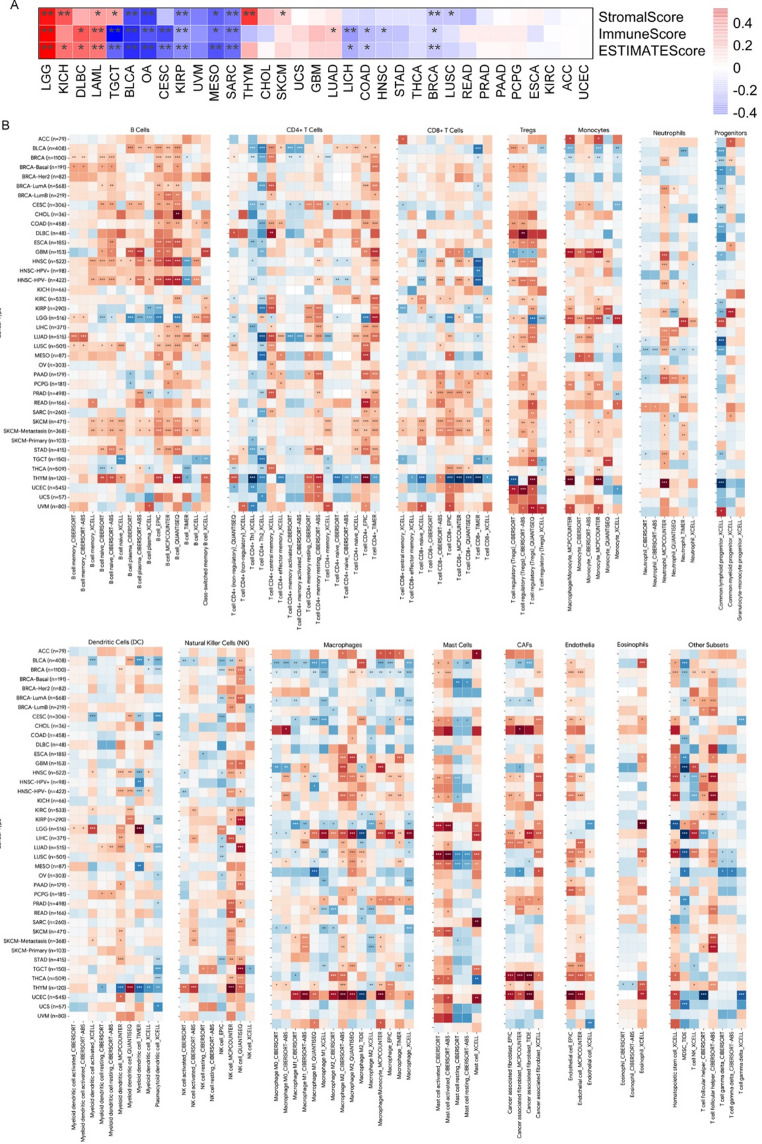
**A** Spearman correlation of *DLEC1* expression and ESTIMATE scores (raw p-values). **B**, **C** Spearman correlation analysis of *DLEC1* expression and immune cell infiltration. The color intensity represents the correlation coefficient (R). Statistical significance was corrected using the Benjamini-Hochberg FDR method, with asterisks denoting adjusted p-values: * FDR < 0.05, ** FDR < 0.01, and *** FDR < 0.001

We assessed the correlation of immune microenvironment and *DLEC1* expression through R package *TCGAplot*. As shown in Fig. 5A, immune scores were significantly positively correlated with *DLEC1* expression group in LGG, DLBC, LAML and LUAD, while the immune scores were significantly negative correlated with *DLEC1* expression in TGCT, BLCA, OV, CESC, KIRP, MESO, SARC, LIHC, COAD, HNSC and BRCA. Additionally, we downloaded *DLEC1* expression and immune cell infiltration spearman correlation data from the TIMER2.0 database and visualized it (Fig. 5B-C). *DLEC1* expression showed a significant negative correlation with macrophage, MDSC and γδT cells infiltration and a significant positive correlation with Tregs, B cells, monocytes and TFH cells infiltration (Fig. 5B-C).

#### Prognostic value of *DLEC1* for tumor immunotherapy response

**Fig. 6:**
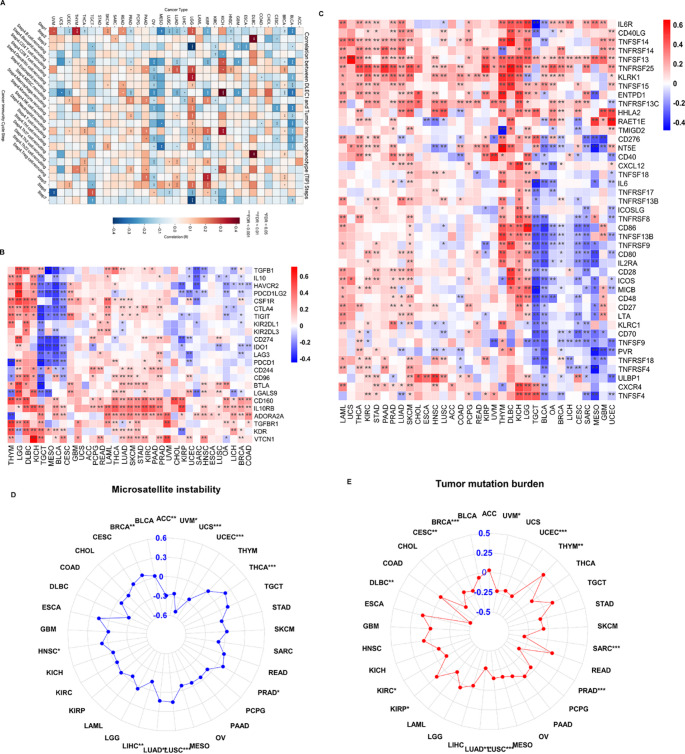
**A** Correlation of *DLEC1* expression with cancer immune scores. The color intensity represents the correlation coefficient (R). Statistical significance was corrected using the Benjamini-Hochberg FDR method, with asterisks denoting adjusted p-values: * FDR < 0.05, ** FDR < 0.01, and *** FDR < 0.001. **B**, **C** Correlation of *DLEC1* and immune-regulate molecules in cancers. **D**, **E** Correlation of *DLEC1* expression with TMB and MSI in cancers. P-values displayed in B-E were raw p-values for exploratory analysis

Given our previous analysis indicating the regulatory role of *DLEC1* expression in immune microenvironment across various human cancers, we further examined the predictive potential of *DLEC1* for cancer immunotherapy response. We evaluated activity scores of the anticancer immune response downloaded from the TIP database. As shown in Fig. 6A, high *DLEC1* expression negatively correlated with cancer cell antigen release (Step 1), priming and activation (Step 3), and killing of cancer cells (Step 7), suggesting an immunosuppressive role of *DLEC1* in TME.

Immune-related regulators play crucial roles in modulating the TME and affecting the efficacy of cancer immunotherapy. We performed heatmap analysis to investigate the correlation between *DLEC1* and immune-related molecules in 33 cancer types. Results indicated that *DLEC1* negatively correlated with immunostimulatory factors in most cancer types, specifically in BLCA, OV, BRCA, SARC, MESO and UCEC. Notably, high *DLEC1* expression levels negatively correlated with various immune checkpoint molecules, including CD274 (PD-L1), TIGHT, PDCD1 (PD-1), CTLA4 and LAG3 in TGCT, MESO, BLCA, CESC, UCEC, SARC, OV, LIHC, BRCA and COAD (Fig. 6B-C).

Considering the importance of TMB and MSI as key biomarkers for immunotherapy, we evaluated the correlation between *DLEC1* expression and TMB/MSI in various cancer types. *DLEC1* expression negatively correlated with high TMB scores in BRCA, CESC, DLBC, KIRC, LUAD, LUSC, PRAD, SARC, UCEC and UVM. Similarly, high MSI scores positively correlated with *DLEC1* expression in BRCA, HNSC, LUAD, LUSC, PRAD and THYM (Fig. 6D-E). These results suggest that *DLEC1* may serve as a potential indicator associated with the efficacy of cancer immunotherapy in relevant cancers.

**Fig. 7:**
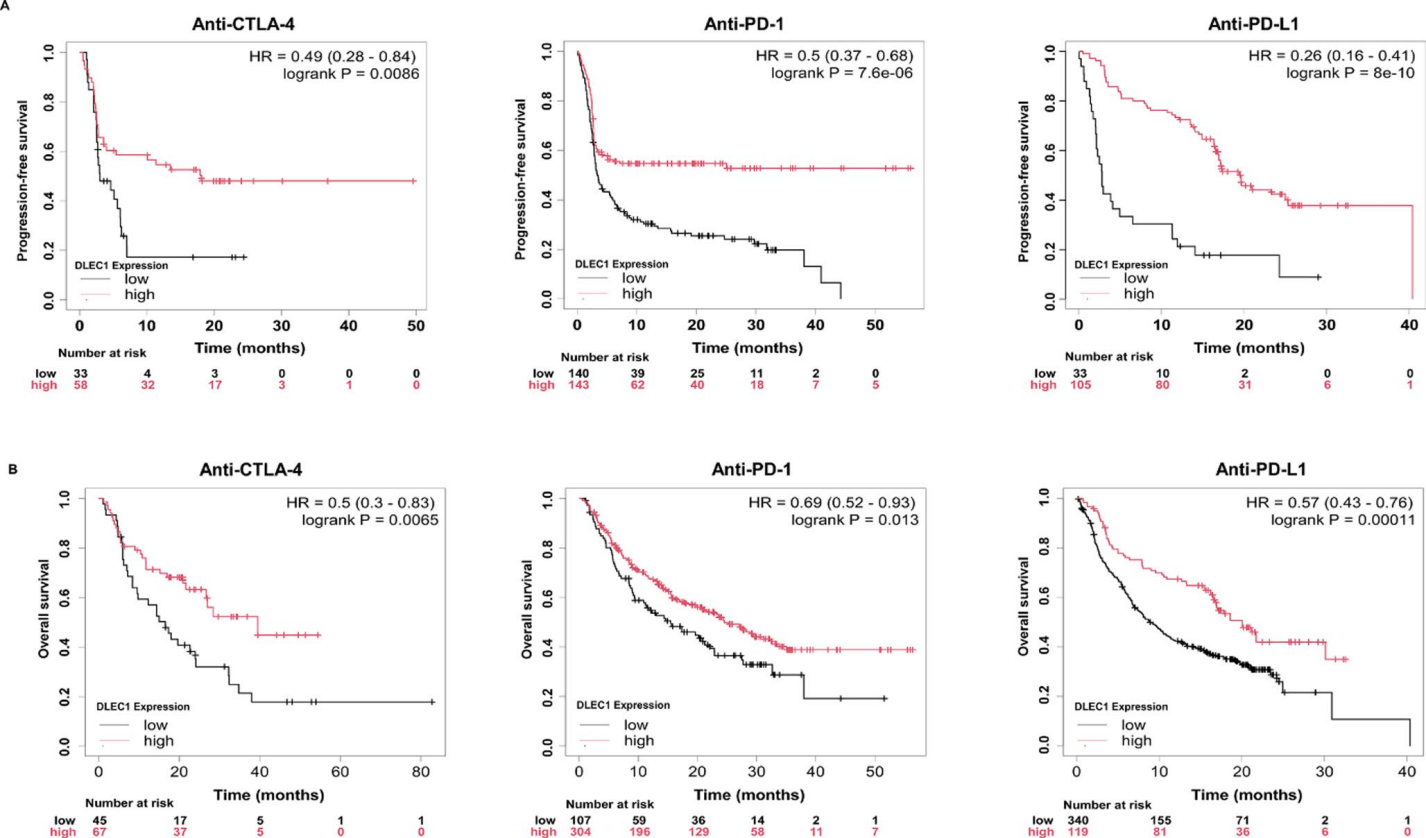
**A**, **B** Survival analysis of cancer patients treated with anti-PD-1, anti-PD-L1, or anti-CTLA-4 immunotherapy in *DLEC1* high and low expression groups

We continued to analyze the prognostic value of *DLEC1* expression as a predictor of immune checkpoint inhibitors therapy. The results indicated that among cancer patients treated with anti-PD-1, anti-PDL-1, or anti-CTLA-4 therapies, those with high *DLEC1* expression showed better prognoses, including overall survival (OS, Fig. 7A) and progression-free survival (PFS, Fig. 7B). These findings reinforced *DLEC1*’s potential as a predictive biomarker for immunotherapy response in cancer.

**Fig. 8:**
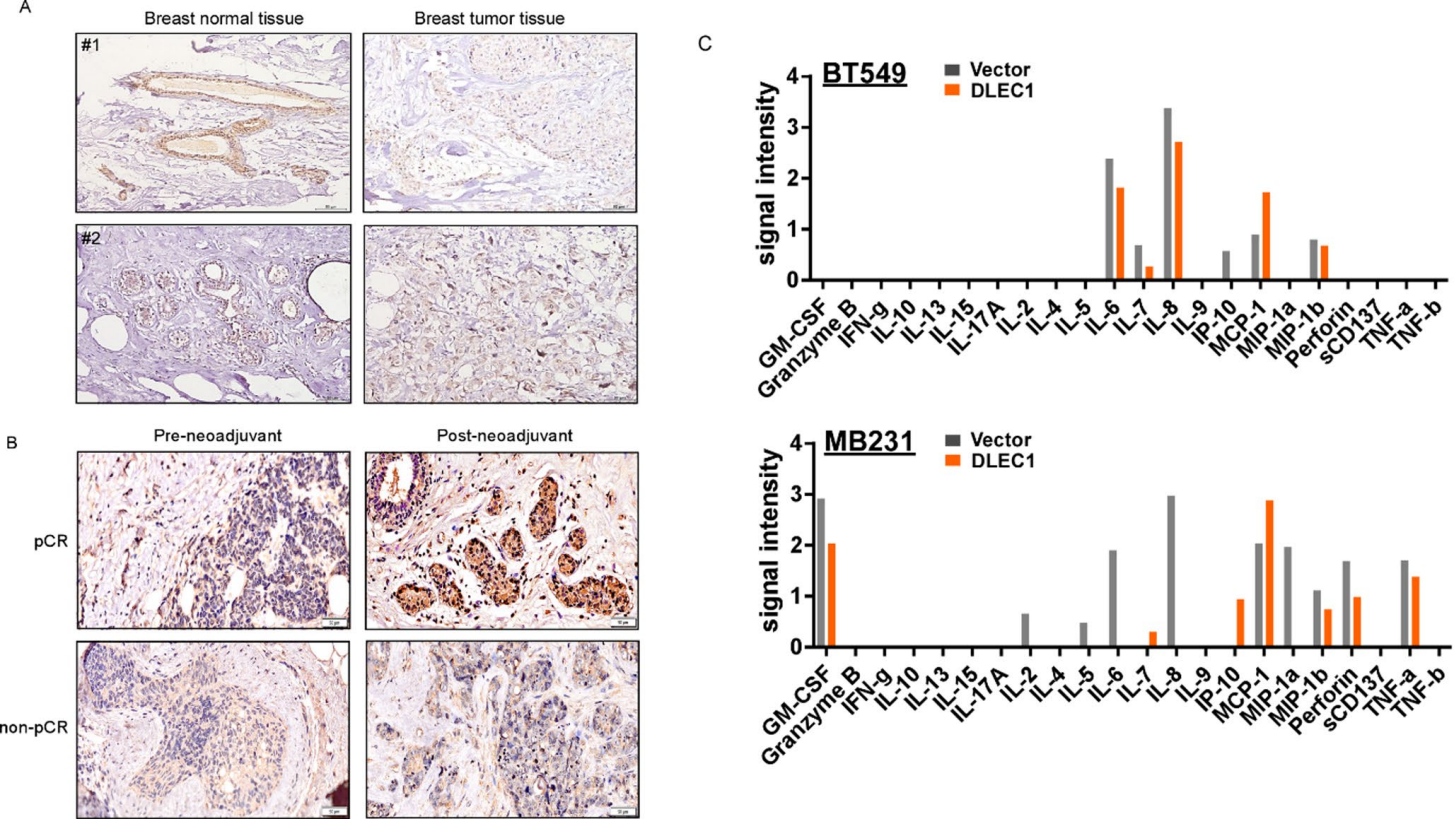
**A** IHC staining for DLEC1 in breast cancer tissues and breast normal tissues. **B** IHC staining for DLEC1 in breast cancer tissues before and after neoadjuvant chemotherapy. **C** Cytokines’ level in cell culture supernatant

Finally, we performed IHC staining for DLEC1 on breast cancer tissues collected before and after neoadjuvant chemotherapy. Positive DLEC1 expression in lymphocytes was observed in pathological complete remission (pCR) patients’ breast cancer tissues, whereas non-pCR patients’ tissue without positive DLEC1 expression in lymphocytes (Fig. 8B). Mechanistically, cytokine chip results showed that restoration of DLEC1 remodeled the immune secretome, specifically reducing the levels of IL6 and IL8 secreted by breast cancer cells (Fig. 8C). These indicated that DLEC1 could affect the immune-related process of breast cancer. These results indicated that DLEC1 may influence breast cancer response to neoadjuvant chemotherapy by modulating the immune microenvironment.

## Discussion

Over the past decade, immunotherapy has proven effective in extending the survival of patients with advanced tumors and continues to revolutionize clinical oncology treatment strategies. Unfortunately, the benefits of immunotherapy are limited to only a subset of patients, primarily due to the heterogeneity of tumor immune microenvironment. Identifying factors/biomarkers that can predict clinical benefit from immunotherapy could improve the selection of responsive tumor types and patient subgroups. In this study, we conducted a pan-cancer analysis and identified *DLEC1* as a potential biomarker for prognosis and immunotherapy response association of multiple human cancers.

In expression analyses, we observed high *DLEC1* expression in actively proliferating normal testis and lower expression in relatively non-proliferative heart and muscle tissues. Analysis of public databases and qRT-PCR results confirmed the low expression and promoter hypermethylation of *DLEC1* in tumor tissues across various cancer types. And we found that this low mRNA level was primarily attributed to promoter hypermethylation of *DLEC1*.

Through OS, DFS, DSS and PFS analyses to predict prognosis, we observed a significant correlation between *DLEC1* expression levels and prognosis in most cancer types. High *DLEC1* expression was identified as a protective factor against tumor occurrence and progression, particularly in breast cancer (BRCA). However, *DLEC1* expression was negatively correlated with good prognosis in cancers such as DLBC, KICH, KIRC, LGG, PCPG, and STAD, indicating a detrimental role in these cancer types. This finding is also supported by published evidence. Overall, these results confirmed that *DLEC1* expression levels could serve as a potential biomarker for predicting the prognosis of patients with various tumors. Notably, despite its high expression inmost types of cancer tissues, DLEC1 appears to play different roles in tumor biology, either promoting or inhibiting tumor growth. Overexpression of DLEC1 significantly inhibited the biological activity of breast cancer cell lines and activated the IFNL1-related pathway. These findings provide a mechanistic link, suggesting that DLEC1 loss may lead to an immunosuppressive phenotype via IFNL1 inactivation.

In analyzing the immunomodulatory functions of *DLEC1*, we found correlations between *DLEC1* expression and immune scores in various tumors, suggesting a significant association with the tumor microenvironment. Further analysis showed that *DLEC1* expression positively correlates with the infiltration of B cells and TFH cells, and negatively correlates with CD4 + T cells, γδT cells, MDSC and NKT cells. TFH cells can promote the growth and differentiation of B cells, thereby facilitating effective adaptive immunity (Vinuesa et al. 2016). Furthermore, TFH cells have been shown to be favorable prognostic factors for cancer patients undergoing immunotherapy across multiple immunotherapy cohorts(Gutiérrez-Melo and Baumjohann 2023). MDSC cells can mediate immunosuppressive responses and promote immune evasion, thereby enhancing tumor progression (Lasser et al. 2024). Additionally, MDSCs can facilitate tumor progression through non-immune pathways, such as promoting angiogenesis, increasing tumor cell stemness, and enhancing epithelial-mesenchymal transition and pre-metastatic niche formation (Safarzadeh et al. 2018, Wang et al. 2019). Moreover, MDSCs have been shown to impair the efficacy of current anti-tumor strategies, including chemotherapy, radiotherapy, and immunotherapy (Li et al. 2021).Additionally, in most cancer types, high DLEC1 levels are negatively correlated with the infiltration of immune cells and the expression of antigen-presenting molecules. These indicated that DLEC1 might play an important role in inhibiting the formation of immunosuppressive microenvironment.

Based on the analysis of patient outcomes following anti-CTLA4/PD-1/PD-L1 therapy, we found that patients with high *DLEC1 *expression had longer survival and recurrence times compared to those with low *DLEC1* expression. These results confirmed the prognostic value of *DLEC1* in determining responses to immunotherapy. In vitro and in vivo experiments also demonstrated that upregulating DLEC1 expression significantly inhibited tumor-like phenotypes in breast cancer cell lines and markedly increased IFNL1 expression. Moreover, in breast cancer patients responsive to neoadjuvant chemotherapy, DLEC1 expression in lymphocytes post-treatment was significantly higher compared to non-responders. Overexpression of DLEC1 also reduced the secretion levels of interleukin-6 (IL6) and interleukin-8 (IL8) in the culture supernatant. Both of these cytokines are associated with immune heterogeneity and can induce the formation of an immunosuppressive microenvironment (Fousek et al. 2021). These results established a potential causal link between DLEC1 expression and the modulation of the breast cancer immune microenvironment, providing a mechanistic explanation for the correlation observed in clinical cohorts. These results suggest that DLEC1 can enhance the efficacy of chemotherapy in breast cancer by influencing the immune microenvironment. Given the crucial role of the immune microenvironment in determining responses to both chemotherapy and immunotherapy, these clinical findings support the bioinformatic predictions that DLEC1 may also possess potential predictive value for immunotherapy.

Tumor treatment regimens, including chemotherapy and radiotherapy, can modulate the immune microenvironment and enhance the effectiveness of these therapies. Chemotherapy can induce immunogenic cell death, which stimulates anti-tumor immunity by promoting the release of tumor antigens and enhancing immune cell infiltration into the tumor microenvironment (Li et al. 2021). Similarly, radiotherapy has been shown to both activate and suppress immune responses, depending on the specific treatment parameters (Demaria et al. 2021). It can enhance the anti-tumor immune response by increasing the infiltration of immune cells such as T cells and natural killer (NK) cells into the tumor site, and by promoting the release of cytokines that modulate immune activity (Sharma et al. 2024). The role of DLEC1 in regulating immune-related pathways and its impact on the tumor microenvironment makes it a significant focus for further research in optimizing cancer treatment strategies.

Previous studies primarily focused on the promoter hypermethylation and loss of *DLEC1* expression in various tumors, highlighting its diagnostic and prognostic value while knowing little about its role, mechanisms, and impact on the immune microenvironment. The low expression of DLEC1 in tumors is often due to promoter hypermethylation and histone hypoacetylation (Kwong et al. 2006). DLEC1 has demonstrated its role in regulating the cell cycle in liver cancer by blocking cells in the G1 phase (Qiu et al. 2008). Further studies suggest that DLEC1 might regulate the cell cycle through the activation of the transcription factor AP-2α, with knockdown of AP-2α showing that DLEC1 promotes tumor cell proliferation (Qiu et al. 2015). Additionally, high DLEC1 expression makes tumor cells less sensitive to 5-FU when AP-2α is knocked down (Qiu et al. 2019). In particular, recent studies showed that DLEC1 could inhibit the phosphorylation of STAT3 in a dose-dependent manner, while YXXQ motif played the key role in the combination of DLEC1 and STAT3 (Li et al. 2018). In the tumor microenvironment, STAT3 plays a pivotal role by creating an immunosuppressive microenvironment that facilitates tumor growth and survival (Zou et al. 2020). It can be activated by various cytokines, such as IL-6 and IL-10, secreted within the tumor microenvironment (Huynh et al. 2017).

Overall, our research demonstrated the prognostic role of *DLEC1* across various cancers and its predictive capacity for immune therapy responses. Additionally, our findings revealed its regulatory function within the immune microenvironment of breast cancer, where overexpression of DLEC1 inhibits the secretion of immune suppressive factors and promotes the expression of immune activating factors. This study contributed to the potential clinical application of DLEC1 as a biomarker for immune therapy responses and as a modulator of immune therapy in breast cancer patients.

We acknowledge that our study has several limitations. First, the predictive value of DLEC1 for immunotherapy response relies primarily on retrospective bioinformatic analyses. Specifically, as the public datasets analyzed primarily consist of immunotherapy-treated cohorts without matched untreated controls or an in-house validation cohort, we could not perform a formal biomarker-by-treatment interaction test. Thus, while DLEC1 is strongly associated with better outcomes in treated patients, future prospective studies with control arms are needed to definitively distinguish its predictive value from potential prognostic effects. Second, regarding the mechanistic depth, although we validated that *DLEC1* expression modulates cytokine secretion and immune microenvironment in vitro, we did not perform in vivo animal studies or rescue experiments (e.g., overexpression/knockdown rescue) to fully dissect the downstream signaling pathways. Consequently, the precise molecular cascade linking epigenetic silencing of *DLEC1* to immune evasion remains to be fully characterized. Third, regarding the immune infiltration analysis, although we applied rigorous FDR correction and adjusted for tumor purity, we could not perform multivariate adjustments for all clinical covariates due to the heterogeneity of clinical annotations in public databases. Future studies utilizing humanized mouse models are warranted to validate these findings in a physiological context.

## Conclusion

In conclusion, our study reveals *DLEC1* exhibits significant promoter hypermethylation and downregulation in most cancer tissues compared to normal tissues. The expression level of DLEC1 holds substantial promise as a valuable biomarker for predicting prognosis and assessing the effectiveness of immunotherapy in human cancers. However, further exploration and verification through additional basic experiments and clinical trials are necessary to elucidate the precise molecular mechanism underlying DLEC1-mediated functions in tumorigenesis and immunotherapy. These future endeavors will enhance our understanding of DLEC1’s role and potential as a therapeutic target in cancer treatment.

## Supplementary Information

Below is the link to the electronic supplementary material.

Supplementary Material 1

Supplementary Material 2

## Data Availability

All data generated or analyzed during this study were included in this published article and its supplementary information files.
