# Supplementary material for: Epigenetic silencing of DLEC1 correlates with tumor immune microenvironment and predicts immunotherapy prognosis in multiple cancers
Source: Hum Genet. 2026 Apr 20;145(1):40. doi: 10.1007/s00439-026-02828-3 (PMC13092530; doi:10.1007/s00439-026-02828-3)
Supplement: Supplementary file 1 — Supplementary Material 1 [file 439_2026_2828_MOESM1_ESM.pdf]

Figure S1: BGS sequencing results of breast cancer and adjacent tissues. BC: breast cancer, BA: breast adjacent tissue, BN: normal breast tissue, BF: breast cancer faraway tissue.

Figure S2: Correlations between mRNA expression level and methylation level of DLEC1 in cancers.

Figure S3: Overexpression of DLEC1 inhibited the malignant phenotype of breast cancer cell lines. (A) Colony formation assays showing reduced colony-forming ability in DLEC1-overexpressing cells. (B) Cell cycle percentage detected by flow cytometry. (C) Flow cytometry analysis showing increased apoptosis in DLEC1-overexpressing cells. (D-E) Transwell assays showing inhibited migration and invasion in DLEC1-overexpressing cells. (F) DLEC1 expression detected by IHC. (G) Ki67 detected by IHC.

Figure S4: Kaplan-Meier survival curves for disease-free survival (DFS) in cancer patients based on DLEC1 expression levels.

Figure S5: Kaplan-Meier survival curves for disease-specific survival (DSS) in cancer patients based on DLEC1 expression levels.

Figure S6: Kaplan-Meier survival curves for progression-free survival (PFS) in cancer patients based on DLEC1 expression levels.

Figure S7: Kaplan-Meier survival curves for post-progression survival (PPS) and first progression (FP) in patients with BRCA, LUAD, and STAD, based on DLEC1 expression levels.

Table S1: Primer sequence.

Table S2. Promoter methylation status of DLEC1 in primary breast tumors

Table S3. DLEC1 methylation and clinicopathologic features of breast tumors.

Figure S1.

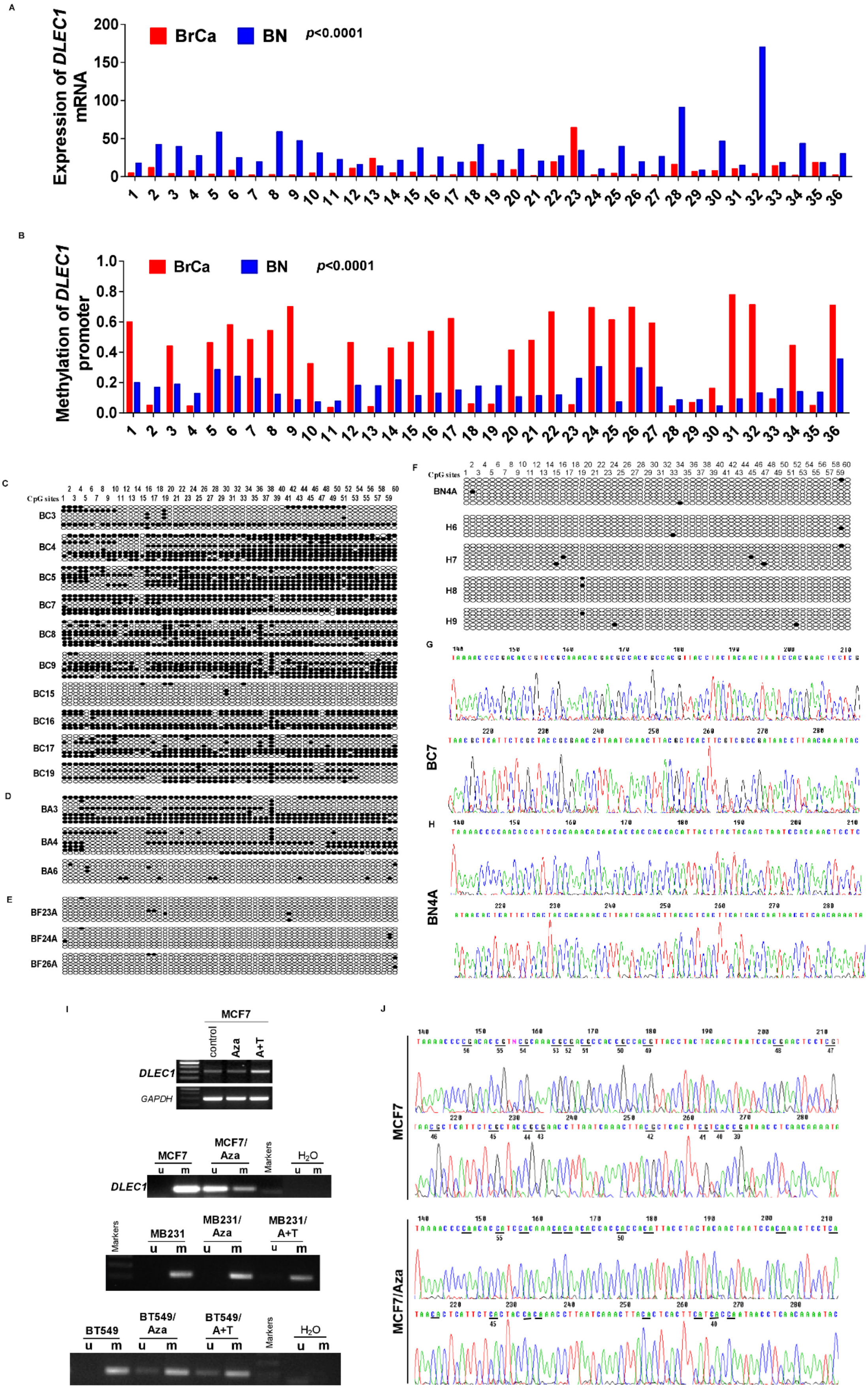
A
Expression of DLEC1 mRNA
BrCa
BN
p<0.0001
B
Methylation of DLEC1 promoter
BrCa
BN
p<0.0001
C
CpG sites
BC3
BC4
BC5
BC7
BC8
BC9
BC15
BC16
BC17
BC19
D
BA3
BA4
BA6
E
BF23A
BF24A
BF26A
F
CpG sites
BN4A
H6
H7
H8
H9
G
BC7
H
BN4A
I
MCF7
control
Aza
A+T
DLEC1
GAPDH
MCF7
MCF7/ Aza
Markers
H2O
u m
DLEC1
MB231
MB231/ Aza
MB231/ A+T
BT549
BT549/ Aza
BT549/ A+T
J
MCF7
MCF7/Aza

Figure S2.

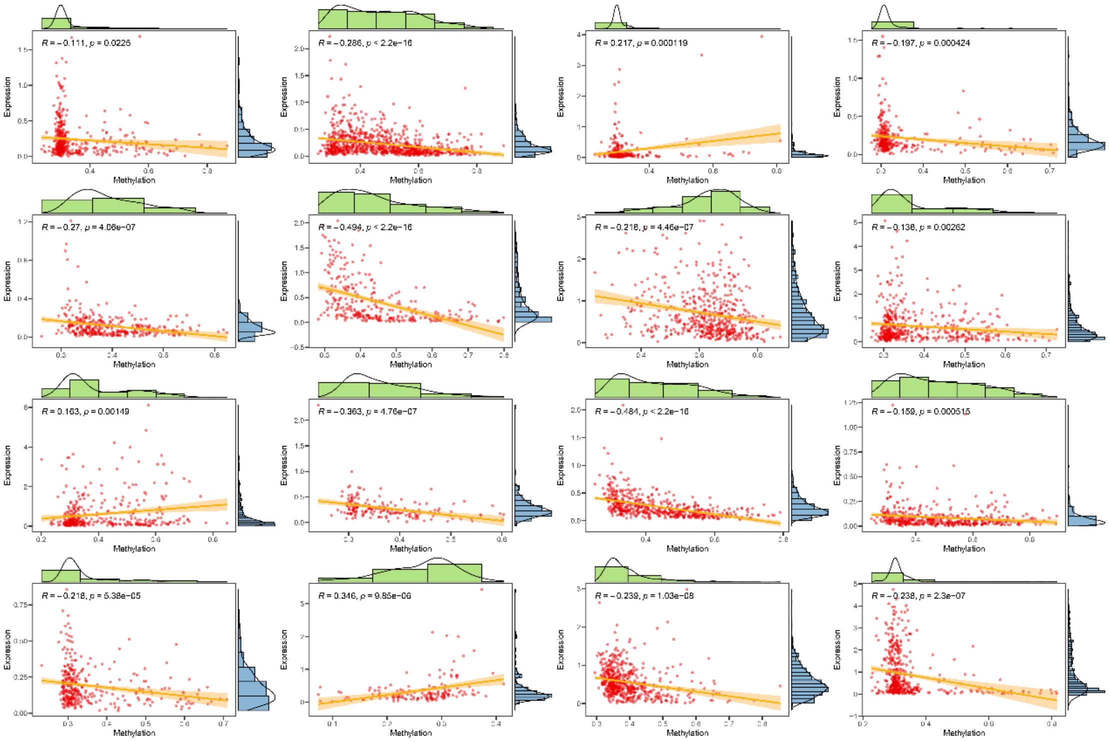
R = −0.111, p = 0.0225
R = −0.286, p < 2.2e−16
R = 0.217, p = 0.000119
R = −0.197, p = 0.000424
R = −0.27, p = 4.06e−07
R = −0.494, p < 2.2e−16
R = −0.218, p = 4.46e−07
R = −0.138, p = 0.00262
R = 0.163, p = 0.00149
R = −0.363, p = 4.76e−07
R = −0.484, p < 2.2e−16
R = −0.159, p = 0.000515
R = −0.218, p = 5.38e−05
R = 0.346, p = 9.85e−06
R = −0.239, p = 1.03e−08
R = −0.238, p = 2.3e−07
Expression
Methylation

Figure S3.

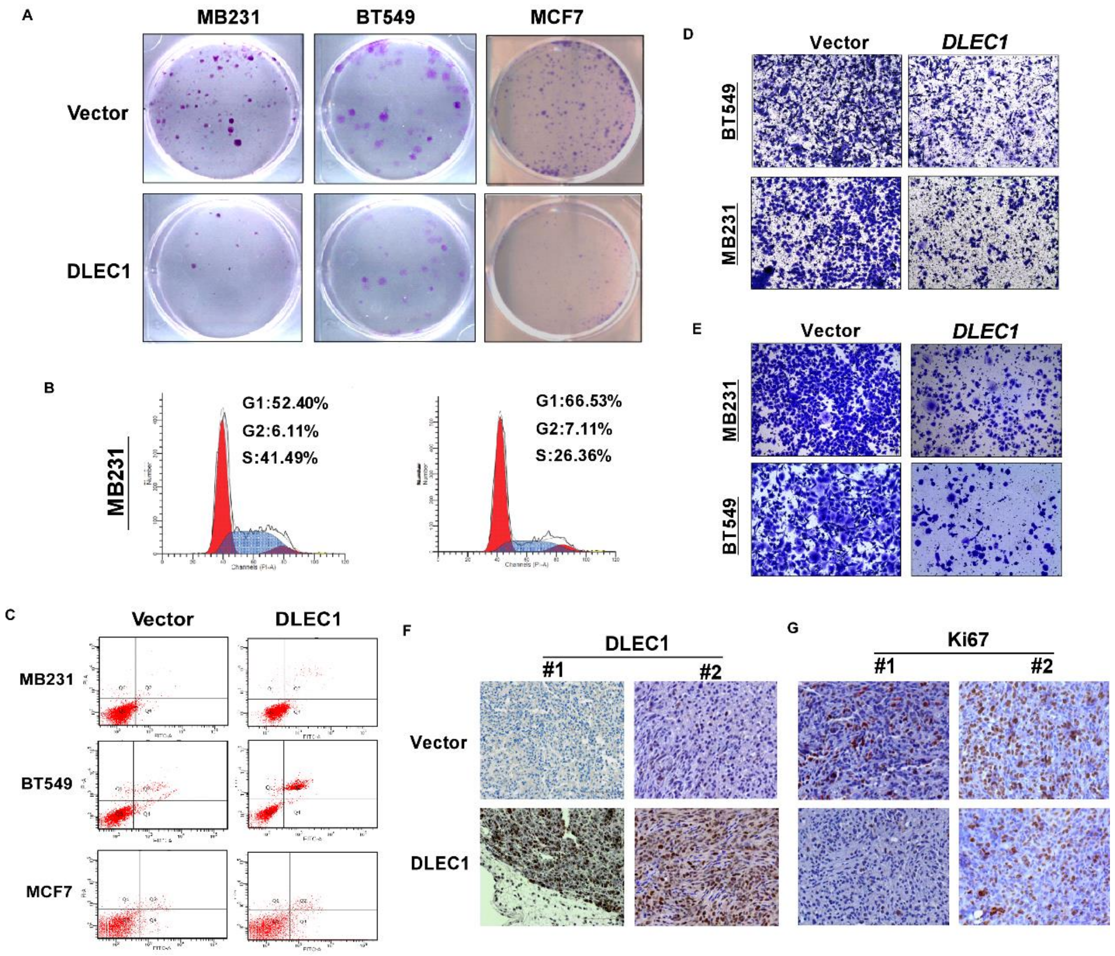
A
MB231
BT549
MCF7
Vector
DLEC1
B
MB231
G1:52.40%
G2:6.11%
S:41.49%
G1:66.53%
G2:7.11%
S:26.36%
C
Vector
DLEC1
MB231
BT549
MCF7
D
Vector
DLEC1
BT549
MB231
E
Vector
DLEC1
MB231
BT549
F
DLEC1
#1
#2
Vector
DLEC1
G
Ki67
#1
#2

Figure S4.

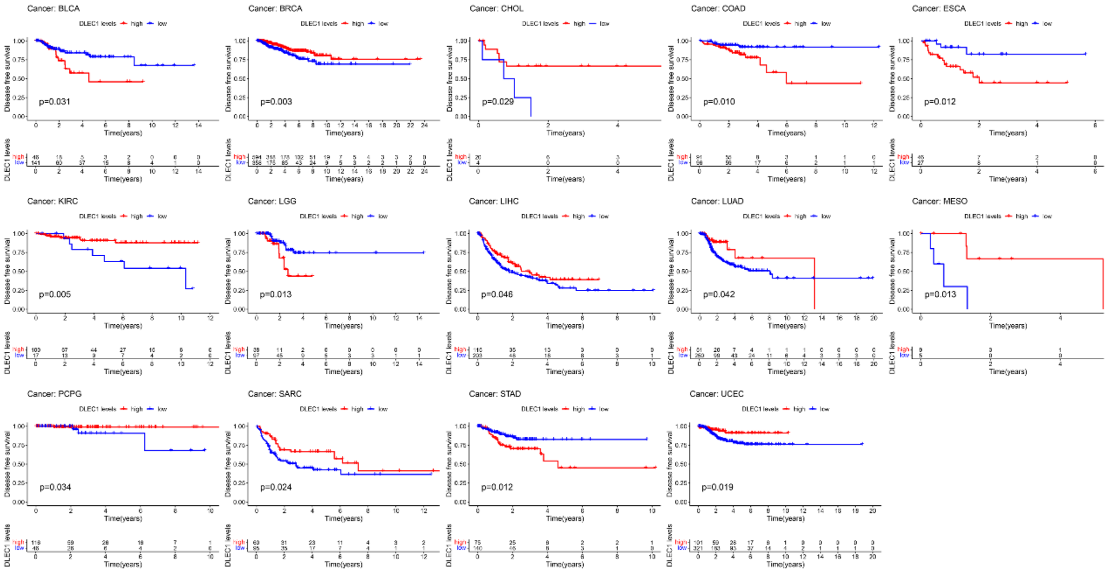

Cancer: BLCA
p=0.031
Cancer: BRCA
p=0.003
Cancer: CHOL
p=0.029
Cancer: COAD
p=0.010
Cancer: ESCA
p=0.012
Cancer: KIRC
p=0.005
Cancer: LGG
p=0.013
Cancer: LIHC
p=0.046
Cancer: LUAD
p=0.042
Cancer: MESO
p=0.013
Cancer: PCPG
p=0.034
Cancer: SARC
p=0.024
Cancer: STAD
p=0.012
Cancer: UCEC
p=0.019

Figure S5.

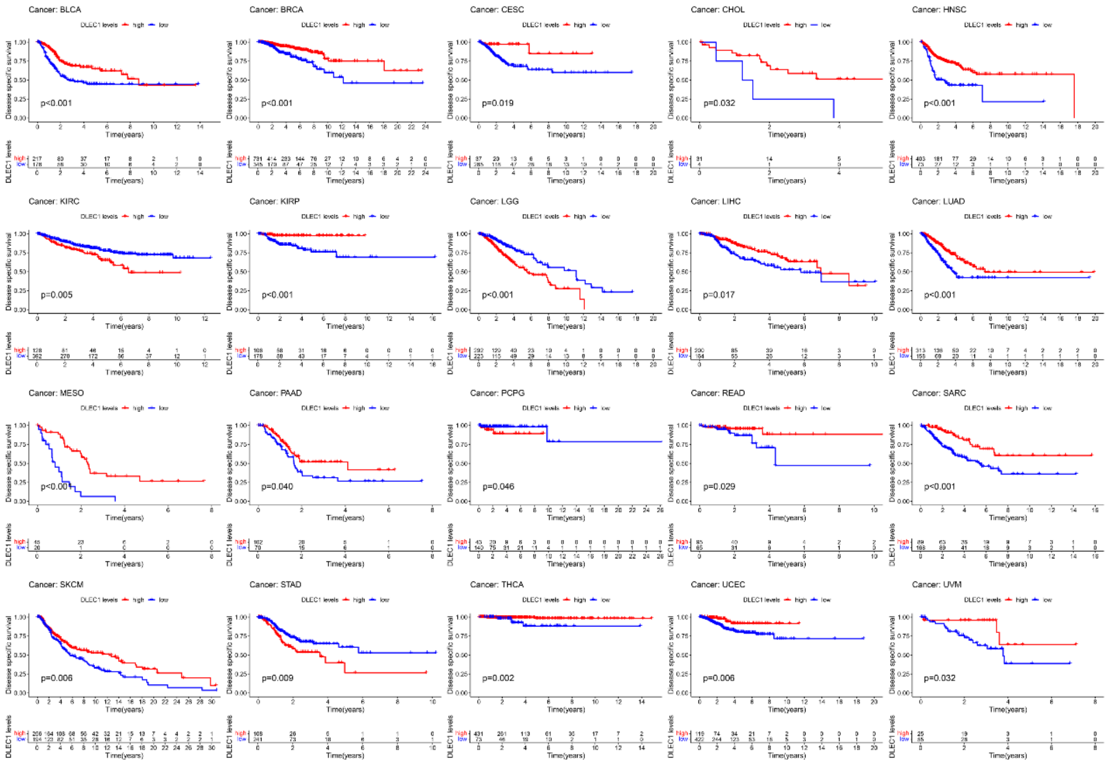

Cancer: BLCA
p<0.001
Cancer: BRCA
p<0.001
Cancer: CESC
p=0.019
Cancer: CHOL
p=0.032
Cancer: HNSC
p<0.001
Cancer: KIRC
p=0.005
Cancer: KIRP
p<0.001
Cancer: LGG
p<0.001
Cancer: LIHC
p=0.017
Cancer: LUAD
p<0.001
Cancer: MESO
p<0.001
Cancer: PAAD
p=0.040
Cancer: PCPG
p=0.046
Cancer: READ
p=0.029
Cancer: SARC
p<0.001
Cancer: SKCM
p=0.006
Cancer: STAD
p=0.009
Cancer: THCA
p=0.002
Cancer: UCEC
p=0.006
Cancer: UVM
p=0.032
DLEC1 levels
high
low
Disease specific survival
Time(years)

Figure S6.

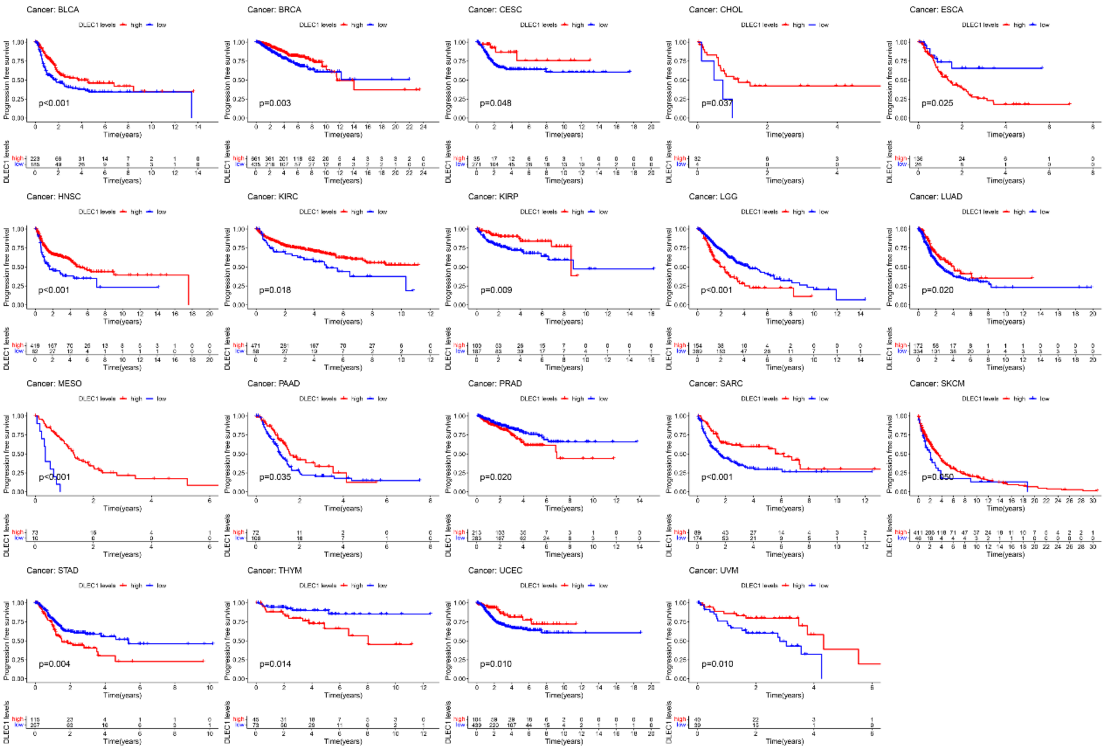

Cancer: BLCA
p<0.001
Cancer: BRCA
p=0.003
Cancer: CESC
p=0.048
Cancer: CHOL
p=0.037
Cancer: ESCA
p=0.025
Cancer: HNSC
p<0.001
Cancer: KIRC
p=0.018
Cancer: KIRP
p=0.009
Cancer: LGG
p<0.001
Cancer: LUAD
p=0.020
Cancer: MESO
p<0.001
Cancer: PAAD
p=0.035
Cancer: PRAD
p=0.020
Cancer: SARC
p<0.001
Cancer: SKCM
p=0.050
Cancer: STAD
p=0.004
Cancer: THYM
p=0.014
Cancer: UCEC
p=0.010
Cancer: UVM
p=0.010
Progression free survival
Time(years)
DLEC1 levels
high
low

Figure S7.

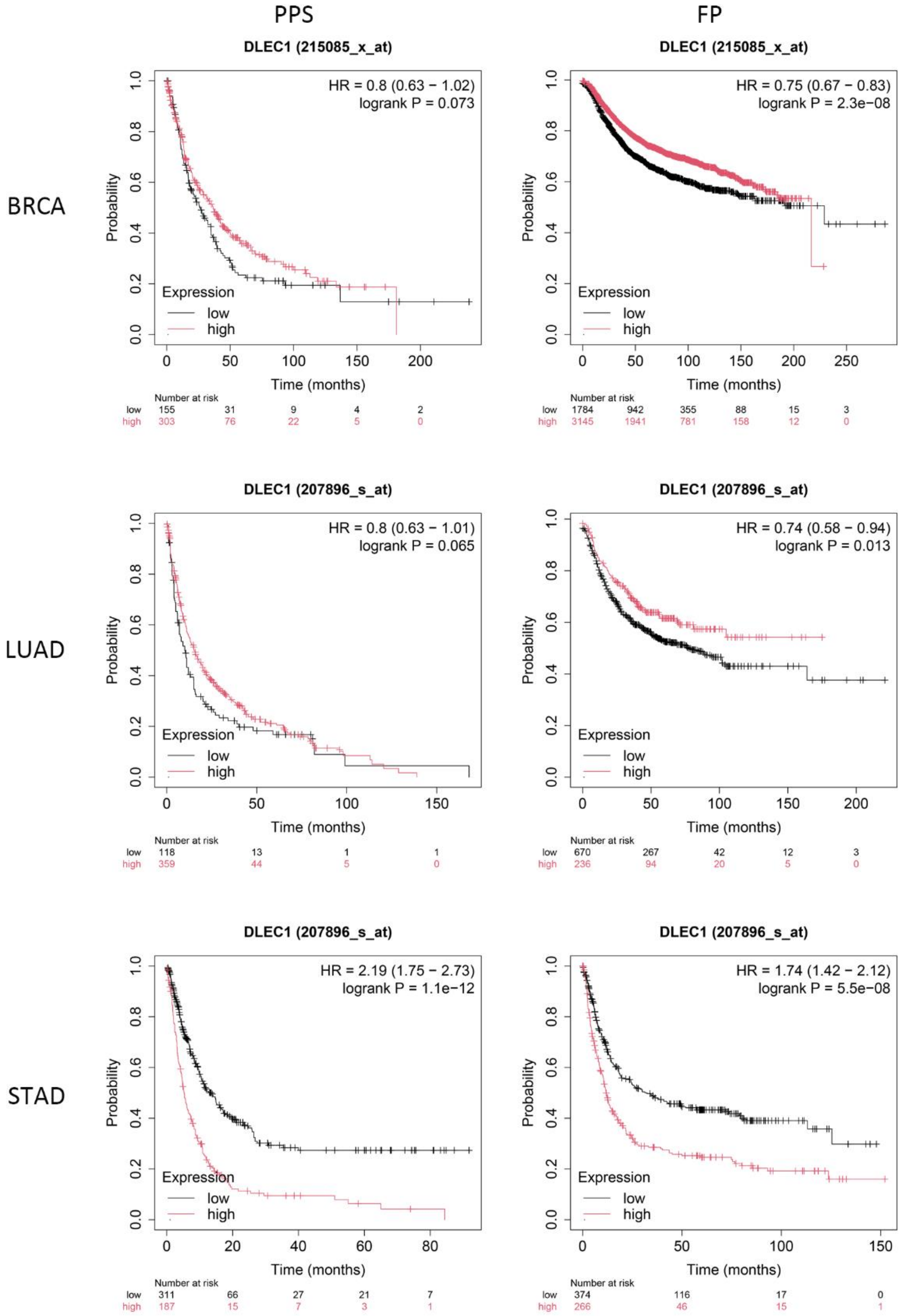
PPS
FP
BRCA
LUAD
STAD
DLEC1 (215085_x_at)
HR = 0.8 (0.63 − 1.02)
logrank P = 0.073
Number at risk
low 155 31 9 4 2
high 303 76 22 5 0
DLEC1 (215085_x_at)
HR = 0.75 (0.67 − 0.83)
logrank P = 2.3e−08
Number at risk
low 1784 942 355 88 15 3
high 3145 1941 781 158 12 0
DLEC1 (207896_s_at)
HR = 0.8 (0.63 − 1.01)
logrank P = 0.065
Number at risk
low 118 13 1 1
high 359 44 5 0
DLEC1 (207896_s_at)
HR = 0.74 (0.58 − 0.94)
logrank P = 0.013
Number at risk
low 670 267 42 12 3
high 236 94 20 5 0
DLEC1 (207896_s_at)
HR = 2.19 (1.75 − 2.73)
logrank P = 1.1e−12
Number at risk
low 311 66 27 21 7
high 187 15 7 3 1
DLEC1 (207896_s_at)
HR = 1.74 (1.42 − 2.12)
logrank P = 5.5e−08
Number at risk
low 374 116 17 0
high 266 46 15 1
Probability
Time (months)
Expression
low
high

Table S1. Primer sequence.

Primer name	Sequence
IFNL1F	CCGTGGTGCTGGTGACTT
IFNL1R	TTTGAACCTGCCAATGTG
IFNL2F	TGCTGGTGCTGATGGC
IFNL2R	GTGGGCTGAGGCTGGA
IFNB1F	TCCTACAAAGAAGCAGCAA
IFNB1R	TCCTCAGGGATGTCAAAG
IFIT1F	AGCCATTTTCTTTGCTTCCC
IFIT1R	ACAGAGCCTTTTCTTCGGTA
DDX60F	GCAAAACCTATGCCTCCTAC
DDX60R	GACGATACTCCCTGGTGAAA
DDX60LF	AAGGGCTTATTAGGGTAGTG
DDX60LR	TTTATTTTGGGCAATGGGAT
OAS1F	CTGACCTGGTTGTCTTCC
OAS1R	GACCTCAAACTTCACGGA
IFIT3F	GGAAACTACGCCTGGGTC
IFIT3R	CACCTTCGCCCTTTCATT
MX2F	TCGTAACCAGGTGTCCGC
MX2R	TACCGATGGTCCTGTCCC
IFI44LF	TATCACCAGCATAACCGAG
IFI44LR	TTGCCAACATTTTAGAGTAGAG
OAS2F	TGAAGCCCTACGAAGAAT
OAS2R	ACTGAAGAAGAGGACAAGG
IFI6F	GGGGTGGAGGCAGGTGAGA
IFI6R	GTGGCGTAGCCCATCAGG
IFIT1F	AGCCATTTTCTTTGCTTCCC
IFIT1R	ACAGAGCCTTTTCTTCGGTA
IFI27F	AAAGTGGTCAGGGTGGC
IFI27R	GGAGGACGAGGCGATT
MX1F	TTGAGATTTCGGATGCTT
MX1R	AGCCACTCTGGTTATGCC
S100A7AF	CCTGCTGACGATGATGAA
S100A7AR	ATGGCTCTGCTTGTGGTA
IFIT3F	GGAAACTACGCCTGGGTC
IFIT3R	CACCTTCGCCCTTTCATT
MX2F	TCGTAACCAGGTGTCCGC
MX2R	TACCGATGGTCCTGTCCC

IFI44LF	TATCACCAGCATAACCGAG
IFI44LR	TTGCCAACATTTTAGAGTAGAG
RSAD2F	CGTGAGCATCGTGAGCAAT
RSAD2R	AATCCCTACACCACCTCCTCA
DLEC1M1	GTTTCGTAGTTCGGTTTCGTC
DLEC1M2	CGAAATATCTTAAATACGCAACG
DLEC1U1	TAGTTTTGTAGTTTGGTTTTGTT
DLEC1U2	ACAAAATATCTTAAATACACAACA
DLEC1BGS1	GAAGATATAAATGTTTATAATGATT
DLEC1BGS2	CAACTACAACCCCAAATCCTAA
DLEC1F1	GATATCTCGCACTTGCTCAC
DLEC1R1	GCTTATAGAGCTCCCGAATCT

Table S2. Promoter methylation status of *DLEC1* in primary breast tumors

Tissue	Samples number	DLEC1 promoter methylated status		Frequency of methylation
		methylated	unmethylated	
Breast tumor	150	130	20	87%
Normal breast	4	0	4	0

Table S3. *DLEC1* methylation and clinicopathologic features of breast tumors

Clinicopathological features	Number （n=140 ）	DLEC1 promoter methylated status		*P* value
		methylated	unmethylated	
**Age**				0.974
≤40	17	15(88%)	2(12%)	
>40	109	95(87%)	15(13%)	
unknown	14	12(86%)	2(14%)	
**grade**				0.635
Ⅰ	9	9(100%)	0	
Ⅱ	88	76(86%)	12(14%)	
Ⅲ	7	6(86%)	1(14%)	
unknown	36	30(83%)	6(17%)	
**Tumour size**				0.27
<2.0 cm	48	43(90%)	5(10%)	
≥2.0 ㎝≤5.0cm	67	59(88%)	8(12%)	
>5.0cm	9	6(81%)	3(19%)	
unknown	16	13(81%)	3(19%)	
**Lymph node metastasis**				0.717
Positive	57	48(84%)	9(16%)	
Negative	67	59(84%)	8(16%)	
unknown	16	13(81%)	3(19%)	
**ER status**				0.606
Positive	61	54(89%)	7(11%)	
Negative	45	37(82%)	8(18%)	
unknown	34	30(88%)	4(12%)	
**PR status**				0.414
Positive	52	44(85%)	8(15%)	
Negative	54	47(87%)	7(13%)	
unknown	34	26(76%)	8(24%)	
**HER2 status**				0.019
>+++	8	6(75%)	2(25%)	
++	49	47(96%)	2(4%)	
<+	49	38(76%)	13(24%)	
unknown	34	30(88%)	4(12%)	
**p53 expression**				0.77
Positive	38	33(87%)	5(13%)	
Negative	50	41(82%)	9(18%)	
unknown	52	44(85%)	10(15%)	
**phase**				0.087
1	39	36(92%)	3(8%)	
2	59	51(86%)	8(14%)	
3	26	22(85%)	4(15%)	
4	1	0	1(100%)	

unknown	15	12 (80%)	3 (20%)
